# Analysis of Alternative mRNA Splicing in Vemurafenib-Resistant Melanoma Cells

**DOI:** 10.3390/biom12070993

**Published:** 2022-07-17

**Authors:** Honey Bokharaie, Walter Kolch, Aleksandar Krstic

**Affiliations:** 1 Systems Biology Ireland, School of Medicine, University College Dublin, Belfield, D04 V1W8 Dublin 4, Ireland; honey.bokharaie@helsinki.fi (H.B.); walter.kolch@ucd.ie (W.K.); 2 Drug Research Program, Faculty of Pharmacy, University of Helsinki, 00014 Helsinki, Finland; 3 Conway Institute of Biomolecular & Biomedical Research, University College Dublin, Belfield, D04 V1W8 Dublin 4, Ireland

**Keywords:** alternative splicing (AS), BRAF (v-Raf murine sarcoma viral oncogene homolog B), malignant melanoma, vemurafenib, drug resistance, cancer, genomics, melanin synthesis, Rho-Rac

## Abstract

**Simple Summary:**

Alternative splicing (AS) is one of the hallmarks of human cancer. One of the most common mechanisms of vemurafenib resistance in malignant melanoma is AS of BRAF, occurring in 15–30% of patients. The aim of our study was to investigate the transcriptome and AS D04landscape in the isogenic BRAF V600E cell line pair SK-MEL-239, where the vemurafenib-resistant derivative expresses a truncated BRAF transcript that lacks the RAS-binding domain. Transcriptome analysis showed differential expression of spliceosome components between the two cell lines. AS analysis, by four different tools, DEXSeq, rMATS, ASpli, and LeafCutter, has identified genes enriched for cell motility and melanin synthesis in vemurafenib-resistant cells. Overlapping predictions for all four tools have been experimentally validated. Our study expands the understanding of melanoma drug resistance from a new perspective and supports the need to investigate in detail the aberrant AS landscape in patients with malignant melanoma.

**Abstract:**

Alternative mRNA splicing is common in cancers. In BRAF V600E-mutated malignant melanoma, a frequent mechanism of acquired resistance to BRAF inhibitors involves alternative splicing (AS) of BRAF. The resulting shortened BRAF protein constitutively dimerizes and conveys drug resistance. Here, we have analysed AS in SK-MEL-239 melanoma cells and a BRAF inhibitor (vemurafenib)-resistant derivative that expresses an AS, shortened BRAF V600E transcript. Transcriptome analysis showed differential expression of spliceosome components between the two cell lines. As there is no consensus approach to analysing AS events, we used and compared four common AS softwares based on different principles, DEXSeq, rMATS, ASpli, and LeafCutter. Two of them correctly identified the BRAF V600E AS in the vemurafenib-resistant cells. Only 12 AS events were identified by all four softwares. Testing the AS predictions experimentally showed that these overlapping predictions are highly accurate. Interestingly, they identified AS caused alterations in the expression of melanin synthesis and cell migration genes in the vemurafenib-resistant cells. This analysis shows that combining different AS analysis approaches produces reliable results and meaningful, biologically testable hypotheses.

## 1. Introduction

Malignant melanoma is a cancer that originates from melanocytes and is ranked 21st among the most common cancers [1,2]. In 2018, 287,723 new cases of melanoma and 60,712 deaths were registered worldwide. Even though melanoma constitutes less than one percent of skin cancer cases, it is highly malignant and responsible for 79% of skin cancer-related deaths [1,2].

Several mutations in melanoma activate signalling pathways that regulate cell proliferation. BRAF, NRAS, and NF1 mutations all activate the MEK/ERK pathway. The MEK/ERK pathway is a signalling cascade that transduces proliferative signals from the extracellular environment to the nucleus of the receiving cells [3]. Normally, the pathway is activated by extracellular ligands, such as growth factors, that bind to receptor tyrosine kinases. These ligand-bound receptors then activate RAS GTPases, which leads to the dimerisation and phosphorylation of RAF protein kinases and the subsequent phosphorylation and activation of the MEK and ERK kinases. Activated ERK can stimulate several transcription factors and regulate genes involved in many cellular processes including cell proliferation. In cancer cells the MEK/ERK pathway is often constitutively activated by mutations, thus promoting the oncogenic transformation of the mutated cells [3]. The most frequent type of mutations in melanoma are mutations in the BRAF oncogene (>60% of cases). In 2002 the cancer genome project identified BRAF mutations in more than 60% of melanomas [4]. BRAF is a serine/threonine-protein kinase, and these BRAF mutations constitutively activate BRAF kinase activity and the downstream ERK pathway [4]. Activation of ERK signalling was confirmed as an early event in human melanoma in 2002 by Cohen et al. [5]. Among the more than 20 different BRAF mutations in melanoma, the BRAFV600E mutation is the most prevalent and accounts for 90% of all BRAF mutations in melanomas.

Because of the prevalence of the BRAF mutations in melanoma, one of the most successful targeted therapies for BRAF mutated melanomas are BRAF kinase inhibitors such as vemurafenib [6]. Like all targeted inhibitors, vemurafenib suffers from the development of resistance leading to patient relapse. In fact, more than 80% of patients experience relapse within eight months of vemurafenib treatment [7]. Resistance mechanisms of BRAF inhibition are chiefly mediated by ERK pathway reactivation, often by directed BRAF alterations, such as BRAF alternative splicing, gene amplification, double kinase fusions, and deletions of the BRAF N terminus [8]. Of those, one of the most common mechanisms in melanoma is the alternative splicing (AS) of BRAF, which occurs in 15–30% of patients [9].

AS is one of the molecular hallmarks of human cancer [10]. Cancer has about 30% more AS events than normal tissue, often producing disease-specific protein isoforms [11,12]. mRNA splicing is mediated by the spliceosome, which is a large complex comprised of five small nuclear ribonucleoproteins U1, U2, U4, U5, U6, and splicing factors including serine and arginine-rich (SR) proteins, heterogeneous nuclear ribonucleoproteins (hnRNPs), and auxiliary proteins [13]. SR proteins regulate splicing by attaching to exonic and intronic splice-enhancer sites, which are sequence motifs within exons and introns [14]. Similarly, hnRNPs regulate splicing by binding to silencer sites that block the access of spliceosome elements and inhibit splicing at these sites [13]. Auxiliary proteins are involved in the assembly of the core-splicing complex to make a functional complex that can produce different splice isoforms from the same gene [13].

Vemurafenib-resistant melanoma cells often express alternatively spliced short BRAF V600E isoforms that lack the RAS-binding domain. In a study of 19 patients that acquired resistance to vemurafenib, four short isoforms were observed in six patients with transcripts lacking exons 4–10, exons 4–8, exons 2–8, or exons 2–10 [15].

A suitable cell model system to study BRAF-splicing mediated vemurafenib resistance are SK-MEL-239 melanoma cells that have acquired resistance [15]. Similar to what was observed in patients, this cell line expresses a short BRAF splice variant that lacks the RAS-binding domain. This splice variant shows enhanced dimerisation, which drastically enhances kinase activity [16,17,18], thus leading to persistent activation of the RAF/MEK/ERK pathway even in the presence of vemurafenib.

Considering that AS is common in cancer, aberrant splicing of BRAF might not be the only splicing event related to vemurafenib resistance. Hence, we sought to characterise systematically the aberrant splicing landscape in vemurafenib-resistant cells.

## 2. Materials and Methods

### 2.1. Cell Culture and Treatments

An SK-MEL-239 clone C3 cell line was received as a generous gift from Prof. Poulikos I. Poulikakos (Department of Oncological Sciences Icahn School of Medicine at Mount Sinai, New York, NY, USA). The establishment of the cell line clone is described by Poulikakos et al. 2011 [15]. The culturing conditions described in the original publication were used. Briefly, parental SK-MEL-239 cells were cultured in RPMI 1640 medium (Gibco, #31870-025, Lot 2425519, Waltham, MA, USA) supplemented with 10% FBS (Gibco, #10270-106, Lot 2166292, Waltham, MA, USA), penicillin/streptomycin (1×) (Gibco, #15140-122, Waltham, MA, USA), and L-glutamine (1×, 2 mM) (Gibco, #25030-024, Waltham, MA, USA). Resistant SK-MEL-239 clone 3 were cultured in the same media supplemented with 2 µM vemurafenib (PLX4032) (SelleckChem, #S1267, Berlin, Germany). Cells were cultivated in cell culture incubator (Thermo Scientific, Waltham, MA, USA) at 37 °C and 5% CO_2_. Depleted medium was replaced with fresh pre-warmed media every two to three days.

### 2.2. Cell Viability Assay

Relative cell viability was measured by MTS assays using the CellTiter 96^®^ AQueous One Solution Cell Proliferation Assay (Promega, #G3581, Lot 0000442059, Madison, WI, USA). Briefly, SK-MEL-239 cells, parental and vemurafenib-resistant clone 3, were seeded in 96-well flat-bottom plates (1 × 10^4^ cells/well) with 100 µL of 10% FCS media and incubated for 24 h. Graded dilutions of vemurafenib (PLX4032) (SelleckChem, #S1267, Berlin, Germany) or DMSO (Sigma, D8418, Darmstadt, Germany) vehicle control, in culture medium, were added to each well in triplicate. Upon drug treatment, MTS reagent was added and, after one hour of incubation, the absorbance at 490 nm was measured using the plate reader (Spectramax Plus384 Plate Reader, Molecular Devices accompanied with SoftMaxPro software, San Jose, CA, USA). The results were background-corrected by subtracting the average signal of wells only containing medium, and normalised to the no-treatment control at the corresponding 48 h and 72 h timepoint. The mean ± standard deviation (SD) of triplicate samples were calculated and plotted against the increasing concentration of vemurafenib treatments (0.078–10 µM) in Microsoft Office Excel.

### 2.3. Western Blot

Parental and vemurafenib-resistant SK-MEL-239 cells were seeded in 6-well plates and allowed to grow for 24 h. Then, culture medium was replaced with fresh medium without drugs or with vemurafenib, at 1 µM or 10 µM concentration. After 30 or 60 min, cells were placed on ice, washed with ice-cold 1× PBS and harvested using 600 µL of lysis buffer (5% NP40, 10 mM Tris-HCl (pH 7.5), 150 mM NaCl) supplemented with protease inhibitor cocktail (cOmplete™ Mini Protease Inhibitor Cocktail, Roche Diagnostics, #11836170001, Basel, Switzerland), and phosphatase inhibitor cocktail (PhosSTOP, Roche Diagnostics, #4906837001, Basel, Switzerland).

Lysates were cleared by centrifugation at 12,000 rpm for 20 min at 4 °C, transferred to fresh tubes and stored at −20 °C. The prepared whole cell lysates were mixed with 4× Loading buffer (44.4% glycerol, 4.4% SDS, 277.8 mM Tris pH 6.8, and 0.02% Bromophenol blue, 100 mM DTT), heated for 5 min at 95 °C, cooled on ice and resolved using SDS-PAGE electrophoresis. The Precision Plus Protein™ Dual Colour ladder (BioRad, #161-0374, Hercules, CA, USA) was used as a molecular weight standard. Upon transfer onto PVDF membrane (Hybond-P, Amersham, #GZRPN303F, Marlborough, MA, USA), membranes were blocked in 5% non-fat milk (Sigma, #70166) in TBST at room temperature for 1 h. Membranes were probed with primary antibodies diluted in 5% (*w*/*v*) BSA in 1× TBST overnight at +4 °C. Next day, membranes were washed and incubated in the horseradish peroxidase (HRP)-conjugated secondary antibodies directed against primary mouse and rabbit antibodies (dilution of 1/5000 in 5% (*w*/*v*) non-fat milk powder in 1× TBST), for 1 h at room temperature. Next, membranes were incubated with ECL substrate (Pierce ECL Western Blotting Substrate, Thermo Fisher, #32106, Waltham, MA, USA) and the chemiluminescent signal was acquired with Chemi Imager (Advanced Molecular Vision accompanied with Chemostar software, London, UK). Primary antibodies directed against the following proteins were used: BRAF (F-7) (1/1000 dilution, #sc-5284, Santa Cruz, Mouse, Dallas, TX, USA), pMEK (1/1000 dilution, #9121, Cell Signaling Technology, rabbit, Danvers, MA, USA), ppERK1/2 (E-4) (1/10,000 dilution, #sc-7383, Santa Cruz, mouse, Dallas, TX, USA), tERK1/2 (1/10,000 dilution, #M5670, Sigma, rabbit, Darmstadt, Germany), pRSK-1/2 (1/1000 dilution, #sc-12898-R, Santa Cruz, rabbit, Dallas, TX, USA), GAPDH (14C10) (1/1000 dilution, #2118, Cell Signalling Technology, rabbit, Danvers, MA, USA). Following horseradish (HRP)-conjugated peroxidase secondary antibodies were used: anti-mouse secondary antibody (1/5000 dilution, #7076, Cell Signalling Technology, horse, Danvers, MA, USA), anti-rabbit secondary antibody (1/5000 dilution, #7074, Cell Signalling Technology, goat, Danvers, MA, USA).

### 2.4. RNA Sequencing

Total mRNA was extracted from four parental SK-MEL-239 and four vemurafenib-resistant SK-MEL-239 RNA biological replicates, using RNeasy Mini Kit (Qiagen, #74104, Lot 163028041, Hilden, Germany) according to the manufacturer’s protocol, and DNA was digested with DNA-free™ DNA Removal Kit (Invitrogen, #AM1906, Lot 00626944, Waltham, MA, USA). RNA integrity was assessed on 2100 Bioanalyzer (Agilent, Santa Clara, CA, USA) using a Eukaryote Total RNA Nano Chip (version 2.6), with samples’ RIN value range ranging from 8.9 to 10. PolyA selection was performed using NEB Next^®^ Ultra ™ RNA Library Prep Kit (New England Biolabs, # E7530L, Ipswich, MA, USA) and the sequencing libraries (250~300 bp insert cDNA library) were generated with a proprietary methodology developed by Novogene, China. We performed 150 bp paired-end sequencing on Illumina NovaSeq 6000 platform (Novogene, Beijing, China).

### 2.5. RT-PCR

We reverse-transcribed 1 µg of the total RNA using the QuantiTect Reverse Transcription Kit (Qiagen, # 205311, Lot 160053800, Hilden, Germany) according to the manufacturer’s protocol. RT-PCR amplification for the detection of selected splice variants was performed for the following genes: *TYR*, *EPB41*, *CLSTN1*, *MPRIP*, *FANCA*, *MARK3*, *EVI5L*, *CAPN3*, *BRAF*, as well as for the housekeeping control gene *GAPDH*. Exon-exon junction spanning primers were designed using Oligo7 (https://www.oligo.net, accessed on 25 April 2022) [19] and optimal design parameters were double checked with Generunner (http://www.generunner.net/, accessed on 25 April 2022) and Primer3Plus (https://primer3plus.com/, accessed on 25 April 2022). Additionally, Primer-BLAST (https://www.ncbi.nlm.nih.gov/tools/primer-blast/, accessed on 25 April 2022) was used to eliminate designed primers with unspecific binding. Primer sequences are provided in the Supplementary Materials Table S6.

PCR was performed in 25 μL reaction volume using the MyTaq Red Mix (Bioline, Meridian Life Science, #BIO-25043, Memphis, TN, USA). The RT-PCR reaction conditions were optimised for each primer pair and are designated as Condition A to D. For Condition A, amplification parameters were: denaturation 1 min at 95 °C, followed by 35 cycles of denaturation at 95 °C for 30 s, annealing at 60 °C for 30 s, and elongation at 72 °C for 10 s, followed by 10 min elongation at 72 °C. For Condition B, amplification parameters were: denaturation 1 min at 95 °C, followed by 30 cycles of denaturation at 95 °C for 30 s, annealing at 59 °C for 30 s and elongation at 72 °C for 10 s, followed by 10 min elongation at 72 °C. For Condition C, amplification parameters were: denaturation 2 min at 95 °C, followed by 30 cycles of denaturation at 95 °C for 30 s, annealing at 61 °C for 30 s and elongation at 72 °C for 40 s, followed by 10 min elongation at 72 °C. For Condition D, amplification parameters were: denaturation 1 min at 95 °C, followed by 35 cycles of denaturation at 95 °C for 30 s, 62 °C for 30 s and 72 °C for 10 s, followed by 10 min elongation at 72 °C. For Condition D, amplification parameters were: denaturation 1 min at 95 °C, followed by 35 cycles of denaturation at 95 °C for 15 s, annealing at 56 °C for 15 s and elongation at 72 °C for 10 s, followed by 10 min elongation at 72 °C. RT-PCR products were separated by electrophoresis in 1% agarose gel (Sigma, # A9539, Darmstadt, Germany). GeneRuler 100 bp DNA ladder (ThermoScientific, #SM0243, Waltham, MA, USA) was used as a marker, and digital images of the gels were taken with MiniBis gel doc system (software GelCapture v7.4; DNR Bio-Imaging Systems, Neve Yamin, Israel).

### 2.6. BRAF Affinity Purification Mass Spectrometry Interactome Analysis

Parental and vemurafenib-resistant SK-MEL-239 cells were seeded in 10 cm Petri dishes and allowed to grow for 24 h, in the presence of 2 µM vemurafenib for resistant cells. Next day, cells were placed on ice, washed with ice-cold 1× PBS and harvested using 1000 µL of lysis buffer (10 mM Tris-HCl (pH7.4), 150 mM NaCl, 10 mM KCl, 1 mM EDTA, 0.1% NP-40) supplemented with protease inhibitor cocktail (cOmplete™ Mini Protease Inhibitor Cocktail, Roche Diagnostics, # 11836170001, Basel, Switzerland) and phosphatase inhibitor cocktail (PhosSTOP, Roche Diagnostics, #4906837001, Basel, Switzerland). Lysates were cleared by centrifugation at 12,000 rpm for 20 min at 4 °C and transferred to fresh tubes. Then, 1 µg of BRAF antibody (F-7) (#sc-5284, Santa Cruz, mouse, Dallas, TX, USA) and of the isotype control (MOPC-21) (Biolegend, #400102, IgG1, mouse, San Diego, CA, USA) were bound to magnetic protein G beads (5 µL per reaction) (Invitrogen, Dynabeads, #10003D, Waltham, MA, USA) in the lysis buffer, with gentle rotation for 90 min at +4 °C. 100 µg of whole cell lysates were used for each immunoprecipitation reaction, with antibody prebound magnetic beads for 2 h and gentle rotation at +4 °C. Beads were washed 3 times with lysis buffer and the final wash was done with 1× PBS. The immunoprecipitates were processed for mass spectrometry as described previously [20]. Mass spectrometry analysis of immunoprecipitates was performed using data-independent analysis parallel accumulation serial fragmentation [21,22]. The mass spectrometer raw data were analysed with DIA-NN 1.8.1, using the library-free search option with the reviewed *Homo sapiens* subset of the reviewed Uniprot Swissprot database [23]. Statistical analysis was performed using Perseus software [24]. Experiments were performed with three biological replicates for each condition. Processed and quantified mass spectrometry data are provided in the Supplemental Table S3.

### 2.7. Ingenuity Pathway Analysis

Functional enrichment analysis was performed by using Ingenuity Pathway Analysis (IPA) (Qiagen, https://www.qiagenbioinformatics.com/products/ingenuitypathway-analysis, accessed on 25 April 2022). First, differentially expressed genes were uploaded to IPA and a pathway enrichment analysis against the Ingenuity Knowledge Base was performed. To identify how many splicing genes were differentially expressed, a BioProfiler analysis for the GO biological function “alternative splicing” was performed. The IPA BioProfiler analysis probes the repository of scientific information to generate molecular profiles of diseases, phenotypes, and biological processes (e.g., alternative splicing), listing all the genes and compounds that have been associated with the profiled term. Additionally, the lists of differentially spliced genes from each splicing analysis tool were uploaded to IPA. The pathway enrichment analysis was performed separately for each tool, then a comparative analysis was performed, for which the tools were treated as multiple conditions. For all analyses, *p*-values for pathway over-representation analysis were generated by IPA using a right-sided Fisher exact test and Benjamini–Hochberg correction for multiple hypothesis testing; *p*-values < 0.05 were considered significant. 

### 2.8. Galaxy Platform

The sequencing quality control analysis was performed on the Galaxy web platform (usegalaxy.org, accessed on 25 April 2022) [25] using FastQC to obtain phred scores for assessing base-calling accuracy and GC content [26]. The paired-end sequence reads (FASTQ files) were aligned to the human reference genome GRCh38 (hg38, GenBank assembly accession: GCA_000001405.28) using HISAT2 aligner [27], using default parameter settings, also on the Galaxy public server. Alignment files were used for the downstream AS analyses.

### 2.9. Biojupies

Differential gene expression analysis was performed on BioJupies web platform (https://amp.pharm.mssm.edu/biojupies/, accessed on 25 April 2022) [28]. The analysis was performed using tools for principal component analysis (PCA), gene clustering (Clustergrammer), differential expression analysis, and volcano plot diagrams. All diagrams were generated using the embedded Plotly tool (https://plot.ly, accessed on 25 April 2022).

### 2.10. Differential Splicing Analysis

Differential splicing analysis was performed using four different tools.

ASpli (Version 2.0.0) is a part of the Bioconductor R package (Release 3.12, doi: 10.18129/B9.bioc.ASpli), and it makes use of junction-reads information and quantifies the pre-mRNA splicing events through calculating PSI and PIR matrix [29]. The AS events with an absolute FDR < 5% and Delta PSI_PIR > 3% were deemed differentially spliced.

DEXSeq (Version1.36.0) is a part of the Bioconductor R package (Release 3.12, doi: 10.18129/B9.bioc.DEXSeq), and it identifies AS through inferring the relative exon usage within each gene [30]. Cut-off criteria were: FDR < 0.05, logFC > 2.

LeafCutter (Version 0.29) was obtained from GitHub (https://github.com/davidaknowles/LeafCutter, accessed on 25 April 2022) and was installed via the R devtools package devtools: install_github (“davidaknowles/LeafCutter/LeafCutter”) [31]. This package identifies AS events by the intron-based clustering approach, where splicing is measured as the excision of introns.

Two packages, rMATS (Version 4.1.0) for differential splicing and Maser (Version 1.7.0), were used for annotating the splicing events with protein domains. rMATS was obtained from the open-source platform SourceForge (http://RNA-seq-mats.sourceforge.net/, accessed on 25 April 2022). Maser was obtained from Bioconductor (Release 3.112) doi: 10.18129/B9.bioc.maser). These two tools are based on quantifying and annotation of exon-included and exon-excluded junction-spanning reads for each AS event [32].

Scripts and parameters are provided in the Supplemental Materials and Methods.

## 3. Results

### 3.1. Characterisation of the Cell Line Model System

**Figure 1:**
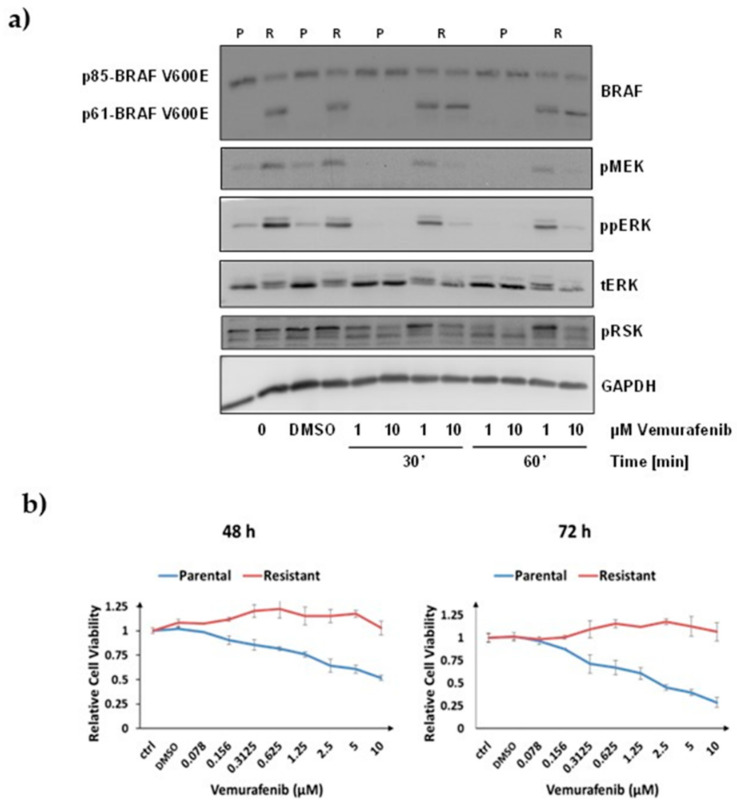
Characterisation of the cell line model system. (**a**) Western blot of ERK signalling in response to vemurafenib treatment. Treatment doses and timepoints in parental (P) and resistant (R) SK-MEL-239 cells are indicated below the image. p85-BRAF indicates full-length BRAF V600E of 85 kDa, and p61 the short splice variant of 61 kDa. (**b**) Relative cell viability measured by MTS assay in response to different doses of vemurafenib treatment for 48 h and 72 h, as indicated. ctrl: No treatment, DMSO: DMSO added, numbers: different concentrations of vemurafenib added as indicated.

For this study we used the human cell-line model system for acquired vemurafenib resistance in malignant melanoma established by Poulikakos et al. [15]. This model system consists of a pair of isogenic human melanoma cells with a BRAF V600E mutation, i.e., parental and drug resistant SK-MEL-239 cells. Vemurafenib-resistant clones were generated from parental SK-MEL-239 cells through continuous long-term drug exposure. Here, we used the resistant clone C3, which expresses a short BRAF splice isoform of 61 kDa in addition to the full-length BRAF isoform of 85 kDa. To assure that the parental cells and Clone 3 respond differentially to vemurafenib, we treated them with 1 µm and 10 µM vemurafenib for 30 min and 60 min and measured the phosphorylation of MEK, ERK, and the ERK substrate RSK1/2 (Figure 1a). In line with the original report by Poulikakos et al. [15], we observed that parental SK-MEL-239 cells expressed the full-length 85 kDa BRAF isoform (p85), while the resistant clone C3 expressed both the p85 full-length and the alternatively spliced 61 kDa BRAF isoform (p61) (Figure 1a). ERK signalling, as assessed by monitoring activating phosphorylation sites in MEK, ERK, and RSK, was completely inhibited by vemurafenib in parental cells under all conditions. By contrast, in resistant cells there was no inhibition with 1 µM of vemurafenib and only partial inhibition with 10 µM vemurafenib (Figure 1a). These results confirmed that the model system has the same characteristics as described in the original report by Poulikakos et al. [15].

To measure the dose- and time-dependent effects of vemurafenib on the viability of SK-MEL-239 cells, we used the MTS cell viability assay (Figure 1b). In parental cells, 10 µM vemurafenib reduced cell viability to 52% and 28% after 48 h and 72 h of treatment, respectively (Figure 1b). In resistant cells, no marked differences of cell viability were observed for any of the drug doses and length of treatment times. These experiments confirmed that parental SK-MEL-293 cells were sensitive to vemurafenib, while the C3 cells were resistant even beyond the 2 μM vemurafenib dose that was routinely included in the growth medium [15].

### 3.2. Eleven Spliceosome Genes Are Differentially Expressed in Resistant Cells

To investigate any changes in transcriptional and AS landscape caused by drug resistance in this model system, we performed RNA-seq in parental and clone C3 SK-MEL-239 cells. RNA-seq data were analysed on BioJupies (https://maayanlab.cloud/biojupies/, accessed on 25 April 2022) and Galaxy (https://usegalaxy.eu/, accessed on 25 April 2022) servers, which enabled us to perform customised analysis with well-established and state of the art RNA-seq pipelines [25,28]. The FastQC quality control analysis [26] on the Galaxy platform showed at least 30 million 150 bp paired-end reads per each of the four biological replicates, with the average phred score of more than 35 across all base pair positions, unbiased and normally distributed GC, confirming the high quality and deep coverage of the RNA-seq data that is important for a reliable AS analysis. Principal component analysis (PCA) showed that 87% of the variance in the RNA-seq data was explained by the first principal component, clearly distinguishing parental and resistant SK-MEL-239 cells (Figure S1). We found 1617 genes were differentially expressed (759 genes were upregulated and 858 were downregulated) at cut-offs of adjusted *p*-value < 0.05 and log2 fold change > 1 (Table S1). Clustering was performed for the top 50 variable genes. The results show two strong clusters of upregulated and downregulated genes that clearly distinguish the parental from the resistant cells (Figure S2).

**Figure 2:**
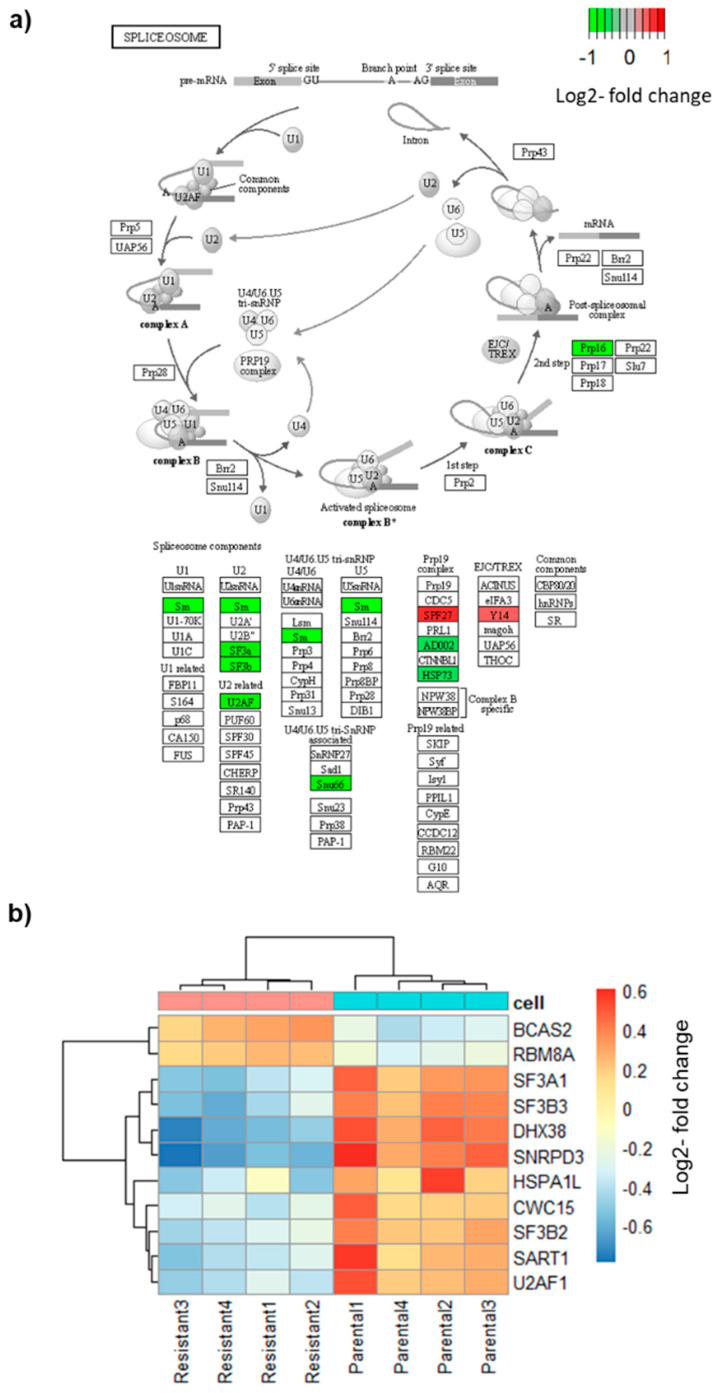
Differential expression of spliceosome genes. (**a**) Scheme of the spliceosome pathway from KEGG (KEGG_SPLICEOSOME, hsa03040). Red indicates downregulated genes, green upregulated gene. (**b**) Heatmap of differentially expressed spliceosome genes.

Because AS of BRAF is the mechanism of resistance in these cells, we examined the genes related to splicing and the spliceosome. First, we looked at all the genes with the GO term “RNA Splicing” (478 genes) and called a gene differentially expressed when the FDR was < 0.05 and the absolute log2 fold change was > 0.5. The results showed that 46 genes were differentially expressed (Table S2). Next, we examined the genes that form the spliceosome as defined by the Molecular Signature Database (KEGG_SPLICEOSOME, hsa03040, 127 genes) [33]. Applying the same cut-off criteria, this analysis revealed that 11 spliceosome genes were differentially expressed, nine were downregulated, and two were upregulated (Figure 2a). The two most differentially expressed genes were *SNRPD3*, a small nuclear riboprotein (Sm) and *DHX38* (*PRP16*), a helicase that participates in the second step in pre-mRNA splicing. Both genes were highly expressed in parental cells and were one log2 fold change downregulated in resistant cells. Three differentially expressed genes, *SPF27* (upregulated), *AD002* (downregulated), and *HSP73* (downregulated), belong to the Prp19 complex which plays a role in splicing, transcription, and mRNA export [34]. Furthermore, three splicing factors, *SF3A1*, *SF3B2*, and *SF3B3*, which belong to the U2 complex, were downregulated in resistant cells, whereas two genes, *RBM8A* (*Y14*) and *BCAS2* (SPF27), were upregulated (Figure 2b). Taken together, a number of genes playing a role at different stages of the spliceosome assembly and mRNA processing, were differentially expressed in vemurafenib-resistant cells.

### 3.3. BRAF Interacts with Spliceosome Components in Parental and Resistant Cells

To investigate the potential interactions of BRAF with spliceosome components and putative changes in the BRAF interactome caused by drug resistance in this model system, we performed BRAF affinity purification mass spectrometry analysis (AP MS) in parental and clone C3 SK-MEL-239 cells. The analysis of the AP MS data revealed the positive enrichment for BRAF interactors, with 97 and 150 proteins for parental and resistant cells, respectively (*p*-value cut-off < 0.05, difference > 0) (Figure S3, Table S3). When we examined proteins that form the spliceosome, as defined by the Molecular Signature Database (KEGG_SPLICEOSOME, hsa03040, 127 genes), we identified 8 BRAF interactors in total, with 6 proteins uniquely interacting with BRAF in resistant cells, 2 interactors common for both parental and resistant cells, and no unique interactors for parental cells (Figure S3). Common interactor FUS (fused-in-sarcoma) couples transcription and splicing, by associating both with RNA polymerase II and the essential splicing factor U1 small nuclear ribonucleoprotein (snRNP) [35], whereas hnRNP C (heterogeneous nuclear ribonucleoproteins C1/C2) promotes the maturation of pre-mRNAs into mRNAs [36]. Among unique BRAF interactors in resistant cells, three are ATP-dependent RNA helicases (SNRPN200, DHX15, DDX15) and together with ALYREF and HNRNPM they are part of the catalytic step 2 spliceosome which occurs upon the first cleavage of the 5′ splice site [37].

### 3.4. Resistant Cells Exhibit Widespread Changes in AS

**Figure 3:**
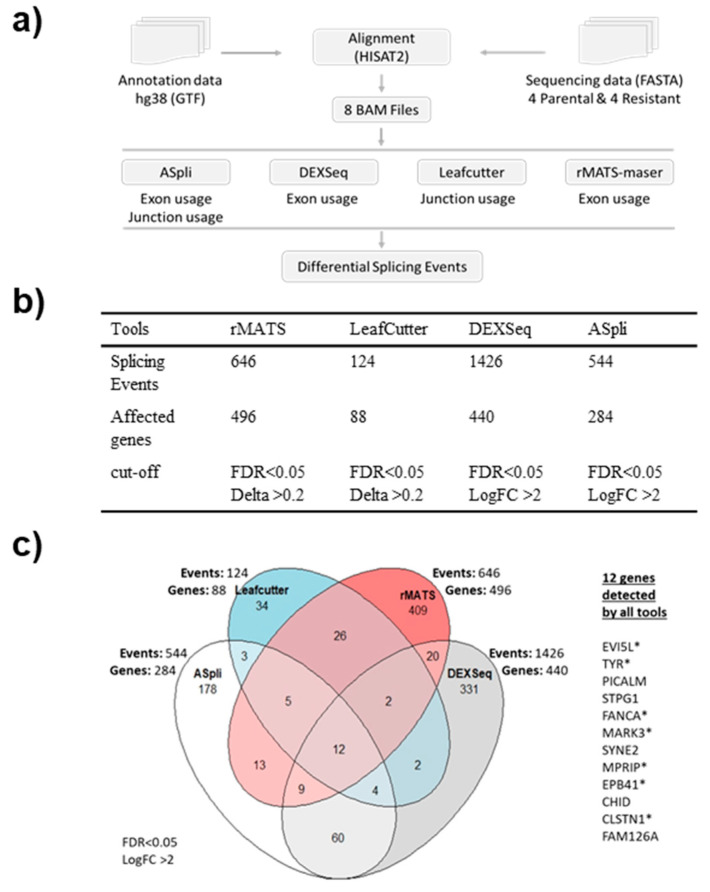
Results of four differential splicing analyses. (**a**) Workflow of the analysis with the four different tools. (**b**) Table showing the number of detected splicing events, affected genes, and cut-offs used for each tool. (**c**) Venn diagram showing the number of splicing events and affected genes for each tool and list of the 12 genes detected by all tools. * indicates the genes selected for further validation.

A consensus on approaches for the differential splicing analysis of RNA sequencing data has not been established yet, with common tools differing substantially in their conceptual approach, statistical analyses, and hence in their output data. Therefore, to perform the differential splicing analysis we employed four different tools that represent three methodological categories: event-based (rMATS-maser [32], LeafCutter [31]), exon usage (DEXSeq [30]), and mixed exon usage and event-based (ASpli [29]) (Figure 3a). The analysis of our RNA-seq data confirmed these observations (Figure 3b). All four tools were used with the same statistical significance cut-offs and identified hundreds of differential splicing events (Figure 3b). DEXSeq identified the most events with 1426, followed by rMATS with 646, ASpli with 544, and LeafCutter with 124 splicing events. The number of differentially spliced genes detected by DEXSeq, rMATS, ASpli, and LeafCutter were 440, 496, 284 and 88, respectively (Figure 3b). All tools call differential exon usage, whereas ASpli and rMATS also call the type of the splicing event, such as exon skipping and alternative splice sites usage (Table S4). Exon skipping was the most common type of event (646 of 905 for rMATS).

**Figure 4:**
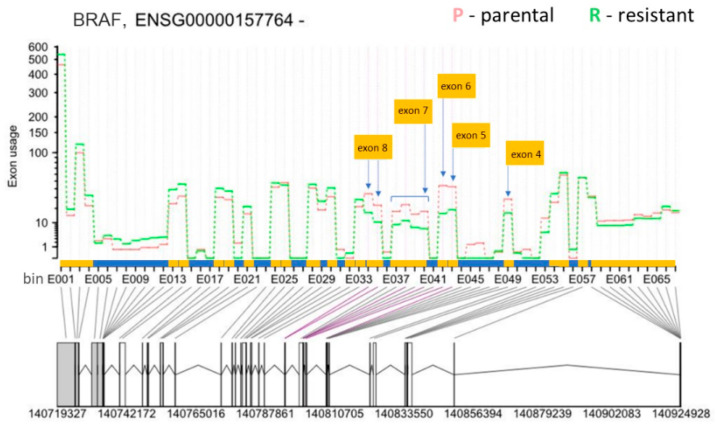
Differential exon usage result for *BRAF* (Ensembl gene ID: ENSG000000157764) from DEXSeq. The *y*-axis shows the exon usage (normalised counts corrected for gene expression) in parental (P) and resistant (R) cells for each exon bin annotated for the plus strand (*x*-axis). Exon bins (E001, …) are sections of the genome that correspond to whole exons or exon fragments in the gene model, as indicated by the grey lines. X-axis is also demarcated for the gene model with exons (yellow boxes) and introns (blue boxes), with exon one starting at the bin number E068 and exon 20 ending at the bin number E001. Below the *x*-axis the gene model is shown with numbers indicating genomic coordinates. Boxes represent exons. Horizontal lines represent introns. The vertical or diagonal lines indicate the position of the exon bins in the gene model. Purple lines indicate statistically significant bin usage. The positions of the skipped exons 4 to 7 in the resistant cells are indicated by yellow boxes and the arrows.

Thus, we focused on differential exon usage to compare all the tools. As a control for the accuracy of the four softwares, we assessed the AS of the BRAF gene that produces the p61 splice form in SK-MEL-239 C3 cells. ASpli and DEXSeq correctly detected the skipping of BRAF exons 4–8 in C3 cells (Figure 4, Table 1) as originally reported by Poulikakos et al. [15], whereas rMATS and LeafCutter did not detect BRAF splicing (Table S4). The observed RAS-binding domain deletion is one of 9 so far identified with BRAF deletions spanning the exons from 2 to 10, with the 2–8 deletion being dominant in melanoma [38]. Interestingly, a systematic study of the *BRAF* AS landscape in melanoma has revealed that *BRAF* is often mutated concurrently with *BRAF* RBD deletions [38].

Apart from *BRAF*, twelve differentially spliced genes were detected by all four tools, suggesting that they are bona fide AS events (Figure 3c). Therefore, we analysed the results for the 12 genes from all four tools in detail. First, we compared the genomic locations of the detected splice junctions. For 11 genes (*EVI5L*, *TYR*, *PICALM*, *FANCA*, *MARK3*, *SYNE2*, *MPRIP*, *EPB41*, *CLSTN1*, *FAM126A*, *CAPN3*) the same splicing events were detected by all four tools. For *CHID1* all four tools detected different events (Table S4).

**Figure 5:**
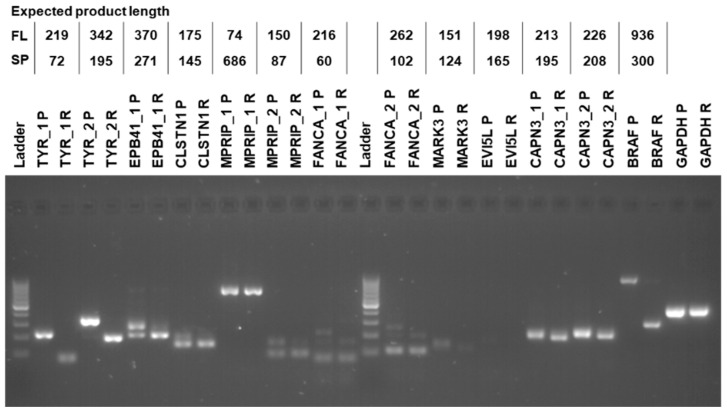
RT-PCR validation. Labels on the top of the gel image provide gene name, primer number in cases when more than one pair of primers were used, parental (P) or resistant (R) sample. Table on top provides the expected RT-PCR product sizes for the full-length (FL) and alternatively spliced (SP) product. Molecular weight DNA ladder marks product sizes from bottom to top: 100, 200, 300, … to 1000 bp.

From the 11 genes detected by all four tools, we chose 7 genes for experimental validation of bioinformatically identified AS events, based on their potential association with melanoma and cancer. We also included *CAPN3*, although AS was only detected by three tools, because of its association with cisplatin resistance and melanoma aggressiveness [39]. For the experimental AS analysis, we designed primers for sequences in the upstream and downstream exons of the exon that was skipped (Table S5). Expected PCR products would differ in size depending on whether the exon was retained or not (Figure 5). For example, for *TYR* we designed two pairs of primers. For both pairs of primers, we detected the long PCR product only in the parental cells and the short PCR product only in the resistant cells (Figure 5). The experimental results fully validated the bioinformatics analysis, confirming that drug resistance of SK-MEL−239 C3 cells is accompanied by specific AS events.

**Figure 6:**
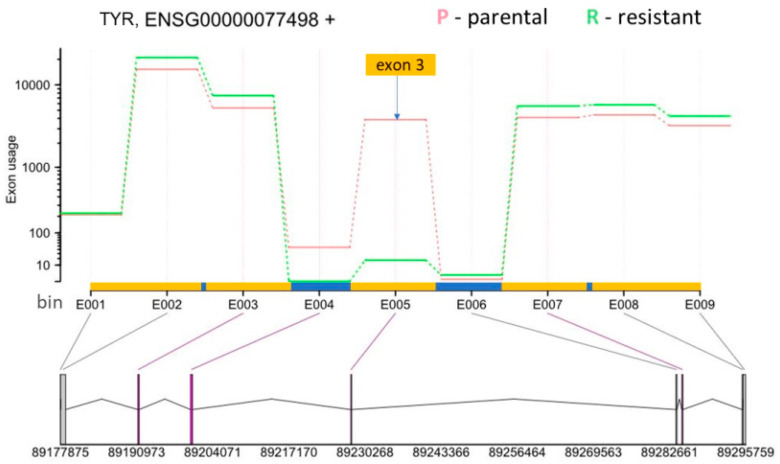
DEXSeq results for *TYR* (Ensembl gene ID: ENSG00000077498). The *y*-axis shows the exon usage (normalised counts corrected for gene-expression) in parental (P) and resistant (R) cells for each exon bin annotated for the plus strand (*x*-axis). Exon bins (E001, …) are sections of the genome that correspond to whole exons or exon fragments in the gene model, as indicated by the grey lines. X-axis is also demarcated for the gene model with exons (yellow boxes) and introns (blue boxes), with exon one starting at the bin number E001 and exon 5 ending at the bin number E009. Below the *x*-axis the gene model is shown with numbers indicating genomic coordinates. Boxes represent exons. Horizontal lines represent introns. The vertical or diagonal lines indicate the position of the exon bins in the gene model. Purple lines indicate statistically significant bin usage. The position of the skipped exon 3 in the resistant cells is indicated by yellow box and the arrow.

One of the most interesting genes that was alternatively spliced is tyrosinase (*TYR*), which is an essential enzyme in melanin synthesis [40]. DEXSeq analysis revealed that a *TYR* exon 3 located on chromosome 11 from 89,227,823 to 89,227,970 (148 bp) was skipped in resistant SK-MEL-239 C3 cells (Figure 6). The skipped exon showed >4000-fold reduction in C3 cells, suggesting an almost complete loss of *TYR* mRNA containing this exon. To deduce the functional consequence of this splicing event, we inspected the data for an overlap between functional protein domains and this splicing event. For this, we used the Ensembl genome browser, which shows functional protein domains and their locations by using functional domain annotations from databases such as pfam, prints, superfamily, and PROSITE (pfam.xfam.org, supfam.org, prosite.expasy.org, respectively). The Ensemble analysis showed two copper-binding domains in the *TYR* gene (Figure S4). Although domain annotations are somewhat different for pfam, superfamily, and PROSITE databases, the second copper-binding domain partially or fully overlapped with the spliced-out exon. TYR needs copper binding to function and the spliced-out domain results in the loss of TYR catalytic function [41]. This suggest that vemurafenib resistance is accompanied by AS changes that incapacitate melanin synthesis.

### 3.5. AS Events Are Correlated with Rho-Mediated Cell Motility

**Figure 7:**
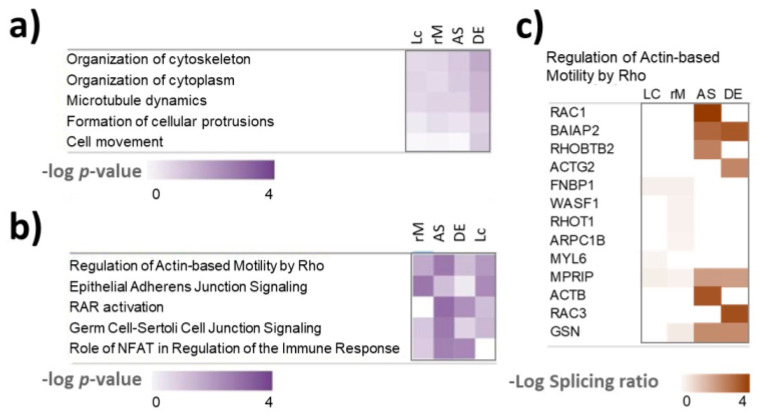
Ingenuity pathway analysis (IPA) of the alternative spliced genes. (**a**) Enrichment of cellular functions. (**b**) Enrichment of canonical pathways. (**c**) Heatmap showing which genes were detected as differentially spliced by the different tools in the actin-based motility by Rho pathway. Lc: LeafCutter; rM: rMATS; AS: ASpli; DE: DEXSeq. Log splicing ratios are absolute values of log ΔPSI for rMATS and LeafCutter and log fold change of exon usage for ASpli and DEXSeq.

To test whether the alternatively spliced transcripts belong to common pathways, we performed IPA analysis on the results for each AS tool and compared the results. The pathway enrichment analysis detected the “regulation of actin-based motility by Rho” pathway as a common pathway for alternatively spliced transcripts identified by all four tools. *MPRIP*, is the only alternatively spliced gene which is detected by all four tools (Figure 7, Table S4). An exon on chr17 from 17,180,607 to 17,180,669 of length 63 bp is skipped in resistant cells. In LeafCutter the junction usage for skipping this exon was 0.368 for parental and 0.42 in resistant cells resulting in a delta PSI 0.368 (Figure S5). MPRIP links Rho signalling to actomyosin contractility [42]. The finding of the “regulation of actin-based motility by Rho” as a common pathway recognised as enriched in the results of four tools (Table S6) is noteworthy, considering that there was limited overlap in the detected differentially spliced genes between the four tools (Table S4). Apart from the AS of *MPRIP*, which was detected by all four tools, different tools identified different alternatively spliced genes in the pathway (Figure 7).

Rho pathway regulating actin-based motility is well known as an important regulator of cancer invasion and metastasis [43,44,45] and has also been linked to BRAF inhibitor resistance in melanoma [44,46]. In the Rho pathway, the Rho-family of GTPases (RhoA, RhoB, and RhoC) function as signalling switches that control myosin-actin dynamics [44]. Rho GTPases can switch from an inactive GDP-bound form to an active GTP-bound form. When active, Rho phosphorylates its target Rho-kinase (ROCK). ROCK then controls myosin light chain (MLC) phosphorylation and activity in two ways. Firstly, ROCK directly phosphorylates myosin light chain (MLC), which controls myosin-actin interactions, stress-fibre contraction and cell motility dynamics [47]. ROCK also deactivates MLC phosphatase, which normally dephosphorylates MLC [48]. Both lead to increased MLC phosphorylation and activity. In this way, activation of the Rho signalling can cause BRAF-inhibitor resistance and was described as a hallmark of therapy resistance in melanoma [44,46].

## 4. Discussion

In this study we demonstrate that wide changes in alternative splicing in melanoma cells occur in the presence of the mutated oncogene BRAF V600E and vemurafenib. Our mRNA-seq analysis has revealed that aberrant splicing coincides with global transcriptome changes, with 1617 DEG (Table S1). Among those DEG, a number of U2 spliceosome complex genes were differentially expressed, with a majority of these genes being downregulated in resistant SK-MEL-239 cells. Switching from one transcript isoform to another is regulated by the levels and activity of RNA binding proteins, either individually or in combination. Isoform switching is regulated by the levels of individual splicing factors or in combination and an important mechanism of alternative splicing deregulation is through alterations in the levels and activity of splicing factors [49]. The complex interplay of the splicing factors is precisely controlled in normal tissue and remains poorly understood. Moreover, the coordinate action of splicing factors in cancer and drug resistance is yet to be understood. Our observation might lead to a conclusion that disrupted gene expression levels, including some splicing factors’ downregulation, in BRAF-driven melanoma cells can result in unbalanced splicing machinery that provides an advantage for cancer cells under a selective pressure of vemurafenib. Although global analysis of cancer-specific variants shows that the variant number per gene is lower than in healthy tissues (1.51 vs. 1.99, respectively), this reduced repertoire is able provide the survival advantage for cancer cells [50].

Our BRAF AP MS data might suggest a novel mechanism of action for BRAF V600E, both for the full-length and truncated isoforms. Namely, to the best of our knowledge, we report for the first time BRAF interactions with spliceosome components. BRAF V600E has been previously identified in the nucleus of some tumour tissues, but the clinical and functional significance of this nuclear staining has never been investigated [51]. However, a recent report by Abd Elmageed et al. demonstrates that nuclear localisation of BRAF V600E is strongly associated with melanoma aggressiveness [52]. Despite the lack of the systematic analyses of the nuclear localisation for truncated BRAF isoforms, our AP MS data suggest qualitative changes in the BRAF interactome, including spliceosome components. It has been already established that splicing factors that control AS are phosphorylated by multiple kinases, including these that specifically phosphorylate serine-arginine- rich proteins (e.g., SR-protein kinases, cdc2-like kinases, topoisomerase 1), and protein kinases that govern key cellular signalling pathways (i.e., AKT) [53]. Phosphorylation of splicing factors regulates their subcellular localisation and interactions with target transcripts and protein partners, with splicing kinases emerging as important regulators of key processes governing malignant transformation, progression, and response to therapeutic treatments [54]. Therefore, we can postulate that both full-length and truncated BRAF kinase is involved in the regulation of the spliceosomal machinery by directly phosphorylating some of the splicing factors and therefore helping in maintaining the splicing landscape that drives the vemurafenib resistance. The specific mechanism of action of the BRAF-spliceosome interactions as well as the role of these interactions in drug resistance remains to be investigated. The complexity of aforementioned changes in the transcriptional landscape and BRAF interactome prompted us to perform a comprehensive analysis of the AS landscape in parental SK-MEL-239 and drug-resistant cells.

For identifying the AS events, we analysed the RNA sequencing data using four different bioinformatics tools. Each tool identified hundreds of AS events, but only 11 splicing events were in common for all four tools (Figure 3, Table S4). This might be explained by the different identification methods used by each tool. ASpli is an R package specifically designed to deal with the possible complexity of splicing patterns, and considers both bin-based signals and junction inclusion indexes, and uses a generalised linear model [29]. Bins are sequences of the genome split into non-overlapping features. Junctions are features connecting one splice-site to another. DEXSeq is also available as an R package and uses a generalised linear model and uses bins to test for differential exon usage and control false-discovery rates [30]. LeafCutter requires SAMtools, Python and R, but avoids the need of transcript annotations and identifies splicing events from short-read RNA sequencing data using a junction-based approach [31]. This approach circumvents the challenges in transcript or exon usage estimation. rMATS requires Python and uses a hierarchical model to simultaneously account for sampling uncertainty in individual replicates and variability among replicates and estimates differential exon usage [32].

The differences in our splicing results show that there is no consensus yet for the analysis of differential splicing. It is well known that different tools use different approaches and therefore recognize different splicing events [55], but how many of these splicing events are false positives is not clear. The implications are twofold. On the one hand, it could mean that the sensitivity of these tools is limited and that different tools recognize different splicing events. On the other hand, it could mean that these tools suffer from false positives. In this case, using several tools and looking at the overlap will reduce the risk of false positives [55]. This was the approach that we have chosen. The result that all seven tested genes could be validated using PCR shows that using several splicing analysis tools and focusing on the overlap is indeed a good approach for minimising false positives. However, it is possible that many of the other identified splicing events are real. For example, LeafCutter does not require genome annotations in terms of known exons, introns and splice sites, and can thus identifies splicing events that cannot be recognised by the other tools [31].

In line with published results, our differential expression analysis shows widespread gene expression changes that distinguish resistant cells [56,57]. A recent study performed RNA-seq analysis in sensitive and resistant A375 melanoma cells found hundreds of differentially expressed genes, but did not analyse alternative splicing [57]. Here, we found differential expression of several splicing factors, including factors of the U2 complex (Figure 2).

Out of the 12 alternatively spliced genes identified by all tools, we have focused our attention on the genes with a putative involvement in the transformation and promotion of the malignant melanoma phenotype. In the following we discuss each of the validated AS events.

One of the alternatively spliced genes with the largest effect size was *TYR* (Figure 6, Figure S4, Table S4). Both, TYR and its binding protein TYRP1 were also top hits for differential expression. Interestingly, while *TYR* expression was slightly downregulated, *TYRP1*, which is involved in the stabilisation of TYR, was upregulated in resistant cells, perhaps as a response to the *TYR* splicing. TYR produces the pigment melanin [40]. Our finding shows that AS of *TYR* in resistant cells causes the loss of the second copper binding domain by exon skipping (Figure 6, Table S4). The two copper binding domains are important for the TYR catalytic function, suggesting a reduction in melanin pigmentation in resistant cells. A previous study showed TYR downregulation and reduced melanin content in vemurafenib-resistant cells consistent with melanoma cell de-differentiation [58]. Similarly, our result suggests reduced TYR activity resulting from AS as a novel mechanism of TYR deactivation in vemurafenib-resistant cells.

*CAPN3* (Calpain 3) AS resulted in the loss of exon 15 in resistant cells (Table S4). The expression of two alternatively spliced short isoforms of *CAPN3* has been observed before in melanoma, and the downregulation of these isoforms has been linked to melanoma aggressiveness and cisplatin resistance [39]. Both of these short *CAPN3* isoforms have exon 15, which contains a nuclear localisation signal [39]. The forced expression of these isoforms induced p53 stabilisation and cell death in A375 human melanoma cells, suggesting that exon 15 is important for the proapoptotic function of CAPN3 [59]. Skipping of exon 15 would mean a loss of the nuclear localisation signal and the proapoptotic function of CAPN3, but because the function of exon 15 is not entirely clear [59], this should be tested in future experiments.

Splicing of *CLSTN1* (Calsyntenin 1) has previously been recognised as very important in tumour invasiveness [60]. Like in many other cancers, the metastatic process of invasive melanoma is driven by the epithelial–mesenchymal transition (EMT), which is characterised by a loss of E-cadherin and a gain of N-cadherin expression. Whereas the expression of E-cadherin (*CDH1*) was not altered in the resistant cells, N-cadherin expression was slightly upregulated (log fold change of 0.3, adjusted *p*-value 0.018, Table S1). In malignant melanoma, EMT enables melanoma cells to cross the basement membrane of the epidermis into the dermis, which is a critical step in the formation of metastases [61]. A CLSTN1 short isoform has been found to inhibit EMT in breast cancer cells [60]. This short isoform lacks exon 11 of the canonical sequence (Ensembl transcript CLSTN1-201, ENST00000361311.4). Here, we identified a short isoform in resistant cells that lacks both exon 11 and exon 3. The findings in breast cancer cells suggest that a lack of exon 11 produces a more epithelial phenotype that is less invasive in the resistant cells. Alternatively, this AS event may enhance the reversion of EMT, which is necessary for cells to proliferate once they have settled into a metastatic site [62]. However, our resistant cells also lacked exon three, and the biological effects of this are not known. Thus, it would be interesting for future work to determine the effects of the here detected splicing events of CLSTN1 on EMT in melanoma cells.

FANCA (Fanconi anaemia complementation group A) is a protein that is involved in the Fanconi anaemia pathway that is activated when DNA replication is blocked due to DNA damage [63]. Germline coding variations and single-nucleotide polymorphism of the *FANCA* gene have been associated with melanoma susceptibility [64] and overall patient survival [65], respectively. Our result that *FANCA* is alternatively spliced suggests alterations of the DNA damage response and repair in vemurafenib-resistant cells.

Of the validated alternatively spliced gens, three genes have not yet been associated with melanoma or vemurafenib resistance.

EPB41 (erythrocyte membrane protein band 4.1), together with spectrin and actin constitutes the cell membrane cytoskeletal network, and plays a key role in regulating membrane mechanical stability and deformability by stabilizing spectrin-actin interaction. The spectrin-actin binding (SAB) domain partially overlaps with the spliced-out exon (ENSE00001065029, exon number 15 in EPB41-201), suggesting that exon skipping results in loss of the EBP41-SAB domain, compromised actin and spectrin binding, and destabilisation of the cytoskeletal network [66].

MARK3 (microtubule affinity regulating kinase 3) is a serine/threonine-protein kinase that phosphorylates the microtubule-associated proteins MAP2, MAP4, and MAPT/TAU [67], negatively regulates the Hippo signalling pathway and cooperates with DLG5 to inhibit the kinase activity of STK3/MST2 toward LATS1 [68]. No known protein domain was associated with the skipped exon (ENSE00003477170, exon number 16 in MARK3-205), making it difficult to speculate about the functional consequence.

EVI5L (ectopic viral integration site 5 like) is a GTPase activating protein (GAP) that modulates cell cycle progression, cytokinesis, and cellular membrane traffic [69]. The functional consequence of the skipped exon (ENSE00002211040, exon number 12 in EVI5L-202) is unknown.

The question of what causes the AS events in drug-resistant cells is still to be answered. Here, we found downregulation of several splicing factors, including *SF3A1*, *SF3B2*, *SF3B3*, *SNRPD4* (SM protein), and *U2AF1*, which are part of the U2 complex (Figure 2). Their downregulation in resistant cells might suggest alterations in the recognition and usage of the intronic branch site sequence. The downregulation of these factors might drive the AS in resistant cells. In line with this idea, silencing of the splicing factor SF3B1 was shown to reduce the expression of the short BRAF V600E isoform in the SK-MEL-239 cell line [70]. Although SF3B1 mutations occur in about 20% of uveal melanomas, the here used SK-MEL-239 cell line is SF3B1 wild-type [71]. Johnson et al. have hypothesised that internal deletions, often accompanied by BRAF mutations, my serve as weak oncogenes on their own, but enhance mutant BRAF signalling [38]. It is possible that BRAF internal deletions contribute to vemurafenib resistance in concert with other resistance mechanisms or in concert with deletions of other genes, as we have demonstrated for 12 other genes, that are able to drive and maintain the oncogenic transformation. The better understanding of complexity of the interplay between AS of BRAF with vemurafenib resistance can be assessed through the evaluation of tumour heterogeneity and clonal outgrowth, as reported by Shi et al. [72]. However, aetiology study of the drug resistance driven by aberrant splicing is not possible using the SK-MEL-239 model system.

As mentioned, we found that the Rho pathway might be regulated by AS in vemurafenib-resistant melanoma cells (Figure 7). Different bioinformatic tools identified different AS genes in the Rho pathway, but *MPRIP* was common to all tools (Figure 7). In the Rho pathway, MPRIP functions as follows. MPRIP binds to MLC phosphatase, locating the phosphatase complex to stress fibres, thus promoting the dephosphorylation of phosphorylated MLC [42]. It is possible that the here identified AS event in *MPRIP* impairs this function, meaning that alternatively spliced MPRIP cannot bind and activate MLC phosphatase, thus promoting MLC activity, stress fibre contractility and therapy resistance. It would be interesting to test this hypothesis in future experiments, for example, by perturbing MPRIP using RNA-interference or switching the alternative splicing of MPRIP back to normal using splice-switching oligonucleotides [73,74].

## 5. Conclusions

Alternative splicing of BRAFV600 is a common mechanism for acquired vemurafenib resistance in melanoma. However, the molecular and genetic mechanisms underlying the vemurafenib resistance driven and/or maintained by aberrantly spliced BRAF remains unclear. Deep understanding of the global transcriptional, including alternative splicing, landscape in drug-resistant melanoma will be crucial for the development of new therapeutic strategies.

## Figures and Tables

**Table 1:** *BRAF* differential exon usage results from ASpli and DEXSeq.

**ASpli**								
Gene	Feature ID	log2 Fold-Change	*p*-value	padj	Chr.	Start	End	Exon number in transcript BRAF-201
BRAF	E033	0.389	0.176	0.719	chr7	140,787,548	140,787,584	Exon 9
BRAF	E034	−1.039	0	0	chr7	140,794,308	140,794,415	Exon 8
BRAF	E035	−1.126	0	0.002	chr7	140,794,416	140,794,467	Exon 8
BRAF	E037	−0.969	0.003	0.101	chr7	140,800,362	140,800,384	Exon 7
BRAF	E038	−1.054	0	0.005	chr7	140,800,385	140,800,437	Exon 7
BRAF	E039	−1.041	0.002	0.08	chr7	140,800,438	140,800,462	Exon 7
BRAF	E040	−1.347	0	0.003	chr7	140,800,463	140,800,481	Exon 7
BRAF	E042	−1.456	0	0	chr7	140,801,412	140,801,560	Exon 6
BRAF	E043	−1.173	0	0	chr7	140,807,960	140,808,062	Exon 5
BRAF	E049	−0.799	0.006	0.152	chr7	140,808,892	140,808,995	Exon 4
BRAF	E054	0.442	0.088	0.576	chr7	140,834,609	140,834,703	Exon 3
**DEXSeq**								
Gene	Entrez:bin	Gene_Coordinates	Start	End	length	logFold-Change	*p*-value	bin.FDR
BRAF	673:E034	chr7:140719327-140924928:-	140,794,308	140,794,415	108	−1.28605	6.45 × 10^−11^	2.21 × 10^−8^
BRAF	673:E035	chr7:140719327-140924928:-	140,794,416	140,794,467	52	−1.42894	2.22 × 10^−10^	7.03 × 10^−8^
BRAF	673:E039	chr7:140719327-140924928:-	140,800,438	140,800,462	25	−1.5363	1.14 × 10^−8^	2.79 × 10^−6^
BRAF	673:E040	chr7:140719327-140924928:-	140,800,463	140,800,481	19	−1.60738	9.21 × 10^−9^	2.28 × 10^−6^
BRAF	673:E042	chr7:140719327-140924928:-	140,801,412	140,801,560	149	−1.48849	4.71 × 10^−14^	2.40 × 10^−11^
BRAF	673:E043	chr7:140719327-140924928:-	140,807,960	140,808,062	103	−1.27428	9.73 × 10^−7^	0.000149

## Data Availability

Sequencing data are deposited in ArrayExpress (www.ebi.ac.uk/arrayexpress, accessed on 25 April 2022) and available under accession number E-MTAB-11609.

## Supplementary Materials

The following supporting information can be downloaded at: https://www.mdpi.com/article/10.3390/biom12070993/s1, Figure S1: Principal component analysis (PCA) of RNA-seq data. Figure S2: Clustergram for DEG; Figure S3: Comparison of BRAF splicing interactors Figure S4: Illustration of the TYR transcripts and domains from the Ensembl genome browser; Figure S5: LeafCutter results for MPRIP gene; Supplemental Figures Legends; Table S1: Differentially Expressed Genes; Table S2: Differentially expressed spliceosome genes; Table S3: BRAF interactome MS data; Table S4:Splicing events; Table S5:RT-PCR primers sequences; Table S6: IPA comparison of canonical pathway enrichment for AS tools; Supplemental Materials and Methods.
