# Supplementary material for: Analysis of Alternative mRNA Splicing in Vemurafenib-Resistant Melanoma Cells
Source: Biomolecules. 2022 Jul 17;12(7):993. doi: 10.3390/biom12070993 (PMC9312936; doi:10.3390/biom12070993)
Supplement: Supplementary file 1 [file biomolecules-12-00993-s001.zip › Supplemental Figures Legends.pdf]

Supplemental Figures Legends

Figure S1. Principal component analysis (PCA) of RNA-Seq data. Four biological replicates in parental (blue circles) and four biological replicates in resistant (red circles) cells using BioJupies. PC1, PC2 and PC3 denote the first, second and third principal component. Indicated percentages show the variance explained by this principal component.

Figure S2. Clustergram for DEG. Top 50 variable genes (rows) for four biological replicates in parental and resistant cells (column) were obtained using Clustergrammer (Fernandez et al., 2017) in BioJupies. Colors represent normalized gene expression values.

Figure S3. Comparison of BRAF splicing interactors. Venn diagram showing the overlap of proteins identified as BRAF interactors by affinity purification mass spectrometry in parental and vemurafenib resistant SK-MEL-239 cells and splicing proteins (KEGG database, pathway ID hsa03040). Purple box - overlapping proteins for KEGG list vs. resistant cells. Light coral box - overlapping proteins for KEGG list vs. resistant cells vs. parental cells. The Venn diagram was generated using jvenn tool (http://jvenn.toulouse.inra.fr/app/index.html).

Figure S4. Illustration of the TYR transcripts and domains from the Ensembl genome browser. The red arrow shows which exon is skipped. The skipped exon contains a copper binding domain.

Figure S5. LeafCutter results for MPRIP gene. Top panel, cluster plot for the comparison of the junction usage in parental versus resistant cells. Numbers are PSI (percentage spliced in) values. The table below shows ΔPSI for each junction usage.
