# Supplementary material for: Analysis of Alternative mRNA Splicing in Vemurafenib-Resistant Melanoma Cells
Source: Biomolecules. 2022 Jul 17;12(7):993. doi: 10.3390/biom12070993 (PMC9312936; doi:10.3390/biom12070993)
Supplement: Supplementary file 1 [file biomolecules-12-00993-s001.zip › Supplemental Materials and Methods.pdf]

Supplemental Materials and Methods

Bionformatics Scripts and Analysis Parameters

ASpli scripts

The ASpli analysis was run on the University College Dublin Computing Cluster using the following R script and parameters:

```
# R-script for ASpli analysis

#load required libaries
library(TxDb.Hsapiens.UCSC.hg38.knownGene)
library(GenomicAlignments)
library(ASpli)
#library(EnsDb.Hsapiens.v86)

#source('loadBamChunk.R')
source("createGRangesGenes.R")
source('createGRangesIntrons.R')
source('createGRangesJunctions.intronsByGene.R')
source('hbcounterJunctions.R')

#link genome annotatation database
TxDb <- TxDb.Hsapiens.UCSC.hg38.knownGene
#TxDb <-  makeTxDbFromGFF("Homo_sapiens.GRCh38.99.chromosome.7.gff3")

#override build in package functions
environment(hbcreateGRangesGenes.getGeneCoordinates) <- asNamespace('ASpli')
assignInNamespace(".createGRangesGenes.getGeneCoordinates",
hbcreateGRangesGenes.getGeneCoordinates, ns = 'ASpli')
environment(hbcreateGRangesIntrons) <- asNamespace('ASpli')
assignInNamespace(".createGRangesIntrons", hbcreateGRangesIntrons, ns =
'ASpli')
environment(hbcreateGRangesJunctions.intronsByGene) <- asNamespace('ASpli')
assignInNamespace(".createGRangesJunctions.intronsByGene",
hbcreateGRangesJunctions.intronsByGene, ns = 'ASpli')
environment(hbgetJunctionOverlapGeneData) <- asNamespace('ASpli')
assignInNamespace(".getJunctionOverlapGeneData", hbgetJunctionOverlapGeneData,
ns = 'ASpli')
environment(hbgetGeneGRanges) <- asNamespace('ASpli')
assignInNamespace(".getGeneGRanges", hbgetGeneGRanges, ns = 'ASpli')

#Bin the genome and save result:

start_time <- Sys.time()
features <- binGenome(TxDb)
end_time <- Sys.time()

save.image("R_WS_features.RData")

#Load BAM files and save result

targets <- data.frame(
    row.names = c('PAR1', 'PAR2', 'PAR3', 'PAR4', 'RES1', 'RES2', 'RES3',
'RES4'),
```

```
    bam = c('PAR1.bam', 'PAR2.bam', 'PAR3.bam', 'PAR4.bam', 'RES1.bam',
'RES2.bam', 'RES3.bam', 'RES4.bam'),
    cells = c( 'P', 'P', 'P', 'P', 'R', 'R', 'R', 'R'),
    stringsAsFactors = FALSE
  )

start_time <- Sys.time()
#bam <- loadBamChunk(targets, 10000000, 6)
bam <- loadBAM(targets, 6)
end_time <- Sys.time()
end_time-start_time
system("free -g")
save.image("R_WS_bam.RData")
print("bam saved")

#Count reads and save results:

start_time <- Sys.time()
counts <- readCounts(features, bam, targets, cores=6, readLength=100L,
maxISize=20000 )
end_time <- Sys.time()
end_time-start_time
system("free -g")
save.image("R_WS_counts.RData")
print("counts saved")

#Run differential usage analysis and save result:

start_time <- Sys.time()
du <- DUreport(counts, targets)
end_time <- Sys.time()
end_time-start_time
system("free -g")
save.image("R_WS_du.RData")
print("du saved")

#writeDU(du, output.dir="run3_du_all")

#Run differential junction usage report and save result:

print("running junctionDUReport...")
start_time <- Sys.time()
du <- junctionDUreport(counts, targets, appendTo = du)
end_time <- Sys.time()
end_time-start_time
system("free -g")
save.image("R_WS_du_junctions.RData")
print("du saved")

#writeDU(du, output.dir="run3_du_all_junctions")

#Run discovery AS analysis and save results:

print("running AS analysis...")
start_time <- Sys.time()
as <- AsDiscover( counts, targets, features, bam, readLength = 100L, threshold
= 5, cores = 6)
end_time <- Sys.time()
end_time-start_time
```

```
system("free -g")
save(as, du, counts, targets, features, file = "R_results_du_as.RData")
save.image("R_WS_du_as.RData")
print("as saved")

#Export all results as tables into output folder:

writeAll(counts, du, as, output.dir="run3_all")

print("Done")
```

The following R scripts were also run:

```
hbcreateGRangesGenes.getGeneCoordinates <- function ( binsGRangesList , aTxDb)
{

  mygenes <- names(binsGRangesList)

  mygenesanno <- genes(aTxDb)

  #getfirstseqnames <- function(myGRanges){
  #  myChr <- as.character(seqnames(myGRanges))
  #  return(myChr[1])
  #}

  #tmp <- sapply(binsGRangesList, getfirstseqnames)

  nomatchidx <- length(mygenesanno)+1
  mygenesanno <- c(mygenesanno, nomatchGRangenomatchGRange <-
GRanges("nomatch:0-0"))
  mask <- match(mygenes, mygenesanno$gene_id, nomatch = nomatchidx)

  myGRanges <- mygenesanno[mask]
  geneCoordinates <- as.character(myGRanges)
  #res <- select(aTxDb, keys = mygenes,
  #              columns=columns(aTxDb),
  #              keytype="GENEID") )

  #geneChr    <- as.character( seqnames( genes( aTxDb ) ) )
  #geneStarts <- sapply( start( binsGRangesList ), min )
  #geneEnds   <- sapply( end(   binsGRangesList ), max )

  #geneCoordinates <- paste0( geneChr, ':', geneStarts, '-', geneEnds )

  #browser()

  return( geneCoordinates )
}
```

```
hbcreateGRangesGenes.getGeneCoordinates <- function ( binsGRangesList , aTxDb)
{

  mygenes <- names(binsGRangesList)

  mygenesanno <- genes(aTxDb)

  #getfirstseqnames <- function(myGRanges){
```

```
  #  myChr <- as.character(seqnames(myGRanges))
  #  return(myChr[1])
  #}

  #tmp <- sapply(binsGRangesList, getfirstseqnames)

  nomatchidx <- length(mygenesanno)+1
  mygenesanno <- c(mygenesanno, nomatchGRangenomatchGRange <-
GRanges("nomatch:0-0"))
  mask <- match(mygenes, mygenesanno$gene_id, nomatch = nomatchidx)

  myGRanges <- mygenesanno[mask]
  geneCoordinates <- as.character(myGRanges)
  #res <- select(aTxDb, keys = mygenes,
  #              columns=columns(aTxDb),
  #              keytype="GENEID") )

  #geneChr    <- as.character( seqnames( genes( aTxDb ) ) )
  #geneStarts <- sapply( start( binsGRangesList ), min )
  #geneEnds   <- sapply( end(   binsGRangesList ), max )

  #geneCoordinates <- paste0( geneChr, ':', geneStarts, '-', geneEnds )

  #browser()

  return( geneCoordinates )
}
```

```
hbcreateGRangesJunctions.intronsByGene <- function ( aTxDb, transcripts,
introns.no.dups ) {
  suppressMessages(
    introns.map <- select( aTxDb,
                           keys    = transcripts,
                           keytype = 'TXNAME',
                           columns = 'GENEID' )
  )
  #EDIT HB:
  mask <-  !is.na( introns.map$GENEID )
  introns.map <- introns.map[mask,  ]
  introns.no.dups <- introns.no.dups[ mask ]
  # Agrupa los intrones por gen
  introns.by.gene <- split( introns.no.dups, introns.map$GENEID )

  return ( introns.by.gene )
}
```

```
# .createGRangesJunctions
hbcreateGRangesJunctions <- function( aTxDb ) {

  # --------------------------------------------------------------------------
#
  # Recupera los intrones por transcripto
  introns.no.dups <- .createGRangesJunctions.getUniqueIntrons( aTxDb )
  # Recupera los nombres de los transcriptos para cada intron
  introns.tx.names <- names( introns.no.dups )
  # --------------------------------------------------------------------------
#
```

```
  # ------------------------------------------------------------------------------
#
  # Agrupa los intrones por gen
  introns.by.gene <- .createGRangesJunctions.intronsByGene( aTxDb,
                                                            introns.tx.names ,
introns.no.dups )
  # ------------------------------------------------------------------------------
#

  # ------------------------------------------------------------------------------
#
# Genera las junturas a partir de los intrones
nIntronsByGene <- lengths( introns.by.gene )
geneNames      <- rep( names( introns.by.gene ) , nIntronsByGene )
junctions <- .createGRangesJunctions.getJunctions( introns.by.gene,
nIntronsByGene, geneNames  )
# ---------------------------------------------------------------------------- #

# ---------------------------------------------------------------------------- #
# Modifica el rangos de las junturas
start( junctions ) <- start( junctions ) - 1
end( junctions )   <- end( junctions ) + 1
# ---------------------------------------------------------------------------- #

# ---------------------------------------------------------------------------- #
# Agrega metadatos
mcols( junctions) <- append( mcols( junctions ), DataFrame( locus = geneNames )
)
# ---------------------------------------------------------------------------- #

# ---------------------------------------------------------------------------- #
# Ordena las junturas
junctions <- sort( junctions )
# ---------------------------------------------------------------------------- #

return (junctions)

}

.createGRangesJunctions.getUniqueIntrons <- function ( aTxDb ) {

  introns <- intronsByTranscript( aTxDb, use.names=TRUE )

  introns.ulst <- unlist( introns )

  introns.no.dups <- introns.ulst[ !duplicated( introns.ulst ) ]

  return ( introns.no.dups  )
}

.createGRangesJunctions.intronsByGene <- function ( aTxDb, transcripts,
introns.no.dups ) {
  suppressMessages(
    introns.map <- select( aTxDb,
                           keys    = transcripts,
                           keytype = 'TXNAME',
                           columns = 'GENEID' )
  )
```

```
  #EDIT HB:
  mask <-  !is.na( introns.map$GENEID )
  introns.map <- introns.map[mask,  ]
  introns.no.dups <- introns.no.dups[ mask ]
  # Agrupa los intrones por gen
  introns.by.gene <- split( introns.no.dups, introns.map$GENEID )

  return ( introns.by.gene )
}

.createGRangesJunctions.getJunctions <- function ( intronsByGene,
nIntronsByGene, geneNames  ) {
  junctions          <- unlist( intronsByGene )
  introns.num        <- sprintf( 'J%03d',  unlist( lapply( nIntronsByGene, seq
) ) )
  junction.names     <- paste( geneNames, introns.num, sep = ":" )
  names( junctions ) <- junction.names
  return( junctions )
}
# Fin de .createGRangesJunctions
```

rMATS scripts

rMATS pipeline consists of two steps. A Python script and a R script. The python script requires two text files that specify the bam file locations, and was run as follows:

```
python rMATS-turbo-Linux-UCS4/rmats.py --b1 PAR.txt --b2 RES.txt --gtf
./gencode.v33.annotation.gtf --od HB_test -t paired --readLength 150 --cstat
0.0001 --libType fr-unstranded
```

with the two text files PAR.txt

```
/home/honey/LeafCutter/hon1/PAR1.bam,/home/honey/LeafCutter/hon1/PAR2.bam,/home
/honey/LeafCutter/hon1/PAR3.bam,/home/honey/LeafCutter/hon1/PAR4.bam
```

and RES.txt

```
/home/honey/LeafCutter/hon1/RES1.bam,/home/honey/LeafCutter/hon1/RES2.bam,/home
/honey/LeafCutter/hon1/RES3.bam,/home/honey/LeafCutter/hon1/RES4.bam
```

R script:

```
# R script for rMATS-maser

library(maser)
library(rtracklayer)

path <- "/home/honey/rMATS/rMATS.4.0.2/MATS_result"

#Run maser:
melanoma <- maser(path, c("PAR", "RES"), ftype = "JCEC")

#Filter results:
melanoma_filt <- filterByCoverage (melanoma, avg_reads = 5)
melanoma_top <- topEvents(melanoma_filt, fdr = 0.05, deltaPSI = 0.1)



#Make a figure: example CAPN3
```

```
melanoma_mygene <- geneEvents(melanoma_filt, geneS = "CAPN3", fdr = 0.05,
deltaPSI = 0.1)
print(melanoma_mygene)
plotGenePSI(melanoma_mygene, type = "SE", show_replicates = TRUE)

#load genome annotation:
gtf_path <-
"/home/honey/LeafCutter/annotation_codes/gencode_hg38_v33/gencode.v33.annotatio
n.gtf.gz"
ens_gtf <- rtracklayer::import.gff(gtf_path)

#Map annotations to results:
mygene_mapped <- mapTranscriptsToEvents(melanoma_mygene, ens_gtf)
head(annotation(mygene_mapped, "SE"))

mygene_annot <- mapProteinFeaturesToEvents(mygene_mapped,
c("Domain_and_Sites"), by="category")
head(annotation(mygene_annot, "SE"))

#Make plot:
plotUniprotKBFeatures(mygene_mapped, "SE", event_id = 7742, gtf = ens_gtf,
   features = c("domain", "binding", "act-site", "Zn-fing", "DNA-bind"),
show_transcripts = TRUE)


#Getting the exon sequences:

library ("BSgenome.Hsapiens.UCSC.hg38")

#getting the sequences for one splicing event at the time:
#get event number 14642. This is skipping an exon (SE) in the CAPN3 gene
SE_myevent <- filterByEventId(melanoma_top, "14649", type="SE")

#get the genomic ranges for this event. For exon skipping, there will ge three
ranges: skipped exon, upstream exon, downstream exon)
myGrange <- granges(SE_myevent, type="SE")

#get the sequences for those genomic ranges
mySequences <- getSeq(BSgenome.Hsapiens.UCSC.hg38,myGrange)
mySequences

#write sequences into a text file
write.table(mySequences, 'seq_CAPN3.txt', quote = FALSE, row.names = FALSE,
col.names=FALSE)
```

LeafCutter script

LeafCutter analysis is a shell script that uses a combination of python and R:

```
#Step 1. Converting bams to juncs
for bamfile in `ls ./*.bam`
do
    echo Converting $bamfile to $bamfile.junc
    sh ../scripts/bam2junc.sh $bamfile $bamfile.junc
    echo $bamfile.junc >> test_juncfiles.txt
done

#Step 2. Intron clustering
../clustering/LeafCutter_cluster.py -j test_juncfiles.txt -m 50 -o testPARvsRES
-l 20000

#Step 3. Differential intron excision analysis
../scripts/LeafCutter_ds.R -i 4 -g 4 --num_threads 4 --
exon_file=../annotation_codes/gencode_hg38_v33/genecode_v33_all_exons.txt.gz
testPARvsRES_perind_numers.counts.gz group_file.txt

#Step 4. Plotting splice junctions
../scripts/ds_plots.R -e
../annotation_codes/gencode_hg38_v33/genecode_v33_all_exons.txt.gz
testPARvsRES_perind_numers.counts.gz group_file.txt
LeafCutter_ds_cluster_significance.txt -f 0.8

#Step 5. prepare the results
../leafviz/prepare_results.R --FDR=0.8 --meta_data_file group_file.txt --code
LeafCutter testPARvsRES_perind_numers.counts.gz
LeafCutter_ds_cluster_significance.txt LeafCutter_ds_effect_sizes.txt
../annotation_codes/gencode_hg38_v33/genecode_v33

#Step 6. Visualize the results change the directory here to
leafviz/prepare_results
#just running this part after first run
cd ../leafviz
./run_leafviz.R  ../hon1/leafviz.RData
```

DEXSeq script

The DEXSeq analysis was run using a R markdown script:

````
---
title: "DEXSeq for whole genome"
author: "Honey"
date: "21 April 2020"
output: html_document
---

```{r setup, include=FALSE}
knitr::opts_chunk$set(echo = TRUE)
```

```{r}
library( "DEXSeq" )

countFiles = list.files("./", pattern=".txt$", full.names=TRUE)
basename(countFiles)

gffFile <- 'Homo_sapiens.GRCh38.99.gff'

sampleTable = data.frame(
  row.names = c( "PAR1", "PAR2", "PAR3", "PAR4",
                 "RES1", "RES2", "RES3", "RES4"),
  condition = c("P", "P", "P", "P",
                "R", "R", "R", "R"),
  libType = c( "paired", "paired", "paired", "paired",
               "paired", "paired", "paired", "paired" ) )

#load data
dxd = DEXSeqDataSetFromHTSeq(
  countFiles,
  sampleData=sampleTable,
  design= ~ sample + exon + condition:exon,
  flattenedfile=gffFile )
```



```{r}
#run DEXseq analysis:

dxd = estimateSizeFactors( dxd )

dxd = estimateDispersions( dxd )

plotDispEsts( dxd)

dxd = testForDEU( dxd )

dxd = estimateExonFoldChanges( dxd, fitExpToVar="condition")

#extract results and write into table:
dxr1 = DEXSeqResults( dxd )
dfdxr1 =data.frame (dxr1$groupID, dxr1$pvalue, dxr1$padj, dxr1$log2fold_R_P,
dxr1$genomicData )
````
````

```
write.table (dfdxr1, file = "DEXSeq-table.txt", append = FALSE, quote = TRUE,
sep = "\t",
            eol = "\n", na = "NA", dec = ".", row.names = FALSE,
            col.names = TRUE)

DEXSeqHTML( dxr1, file="DEXSec-wholegenome.html", FDR=0.01,
color=c("#FF000080", "#00ff00") )
```
```
```
