# Supplementary material for: Analysis of Alternative mRNA Splicing in Vemurafenib-Resistant Melanoma Cells
Source: Biomolecules. 2022 Jul 17;12(7):993. doi: 10.3390/biom12070993 (PMC9312936; doi:10.3390/biom12070993)
Supplement: Supplementary file 1 [file biomolecules-12-00993-s001.zip › Table S5 - RT-PCR primers sequences.pdf]

Table S5

Table S5. RT-PCR primers sequences.

Gene Symbol	Primers
TYR	Forward-1: GCTGCCAATTTCAGCTTTAGAAATAC<br>Reverse-1: AAGAGGACGGTGCCTTCG<br>Forward-2: GCTGCCAATTTCAGCTTTAGA<br>Reverse-2: GTCATAGCCCAGATCTTTGGA
EPB41	Forward: GAAGACGAGCCACCTGAGC<br>Reverse: GCCCATTGATGTTAAGAGTTCG
CLSTN1	Forward: CCACCTACCACGGCATAGTC<br>Reverse: GCATCAAAGGGGACATTCTG
MPRIP	Forward-1: ACCCGGCAACTCAGAAACAT<br>Reverse-1: GACTCTCAGGGGTGTGGTTC<br>Forward-2: CCGGCAACTCAGAAACATC<br>Reverse-2: AAGTCCCTGGATTCAAAGAGC
FANCA	Forward-1,2: TCCGAGAGGTGTTGAAAGAG<br>Reverse-1: CCAGGTGTAACCAGTCTTGG<br>Reverse-2: CGTTCAGTATCTGAAAGAGCATCA
MARK3	Forward: CATGAAGCCACACCATTGTC<br>Reverse: AGCCCTCATATCTCCCGTTC
EVI5L	Forward: CTCCTGAAACAGCGGATTG<br>Reverse: CTGTAGCTGCCGGATGGT
CAPN3	Forward-1: GGATCTGTTCTGGTCATCTGG<br>Reverse-1: GCTTTTGCTTATCAGGGCTTG<br>Forward-2: AGGCGATTGGTTTTAGTGGTAG<br>Reverse-2: CTTGTTTTGCCTTTGCCCTC
BRAF	Forward: CCTATGAAGAATACACCAGCAAG<br>Reverse: CTCCATCACCACGAAATCC
GAPDH	Forward: ACCACAGTCCATGCCATCAC<br>Reverse: TCCACCACCCTGTTGCTGTA
