# Supplementary figures and images for: Analysis of Alternative mRNA Splicing in Vemurafenib-Resistant Melanoma Cells
Source: Biomolecules. 2022 Jul 17;12(7):993. doi: 10.3390/biom12070993 (PMC9312936; doi:10.3390/biom12070993)

Figure S1

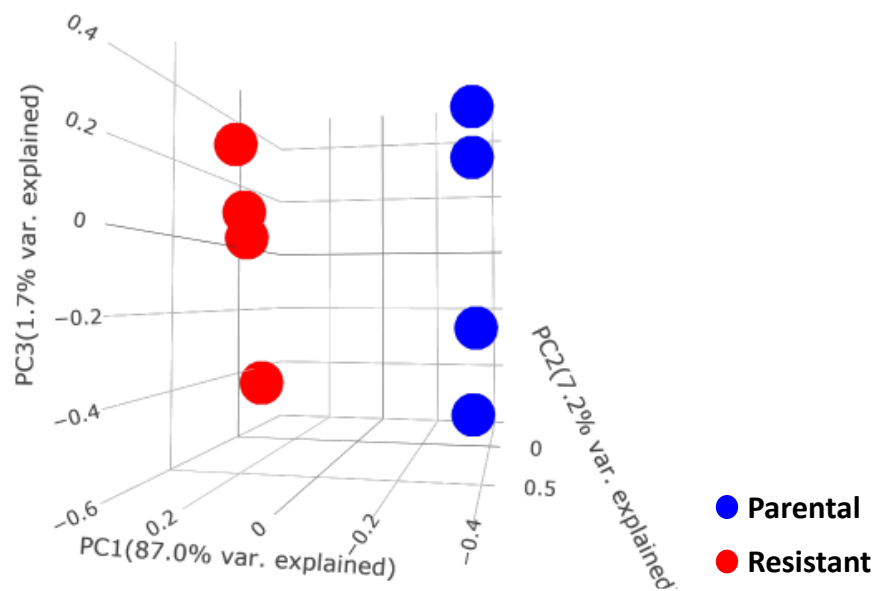

Figure S2

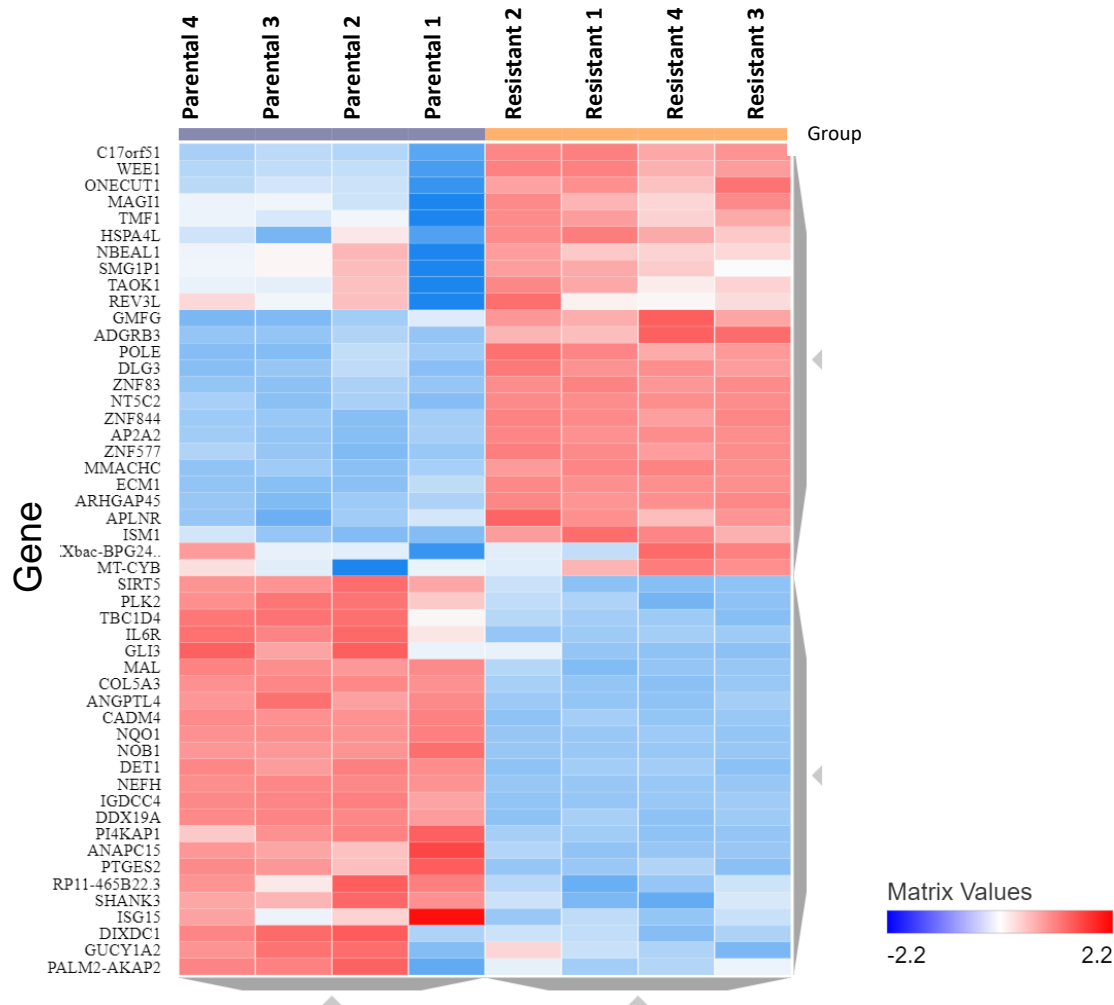

Figure S3

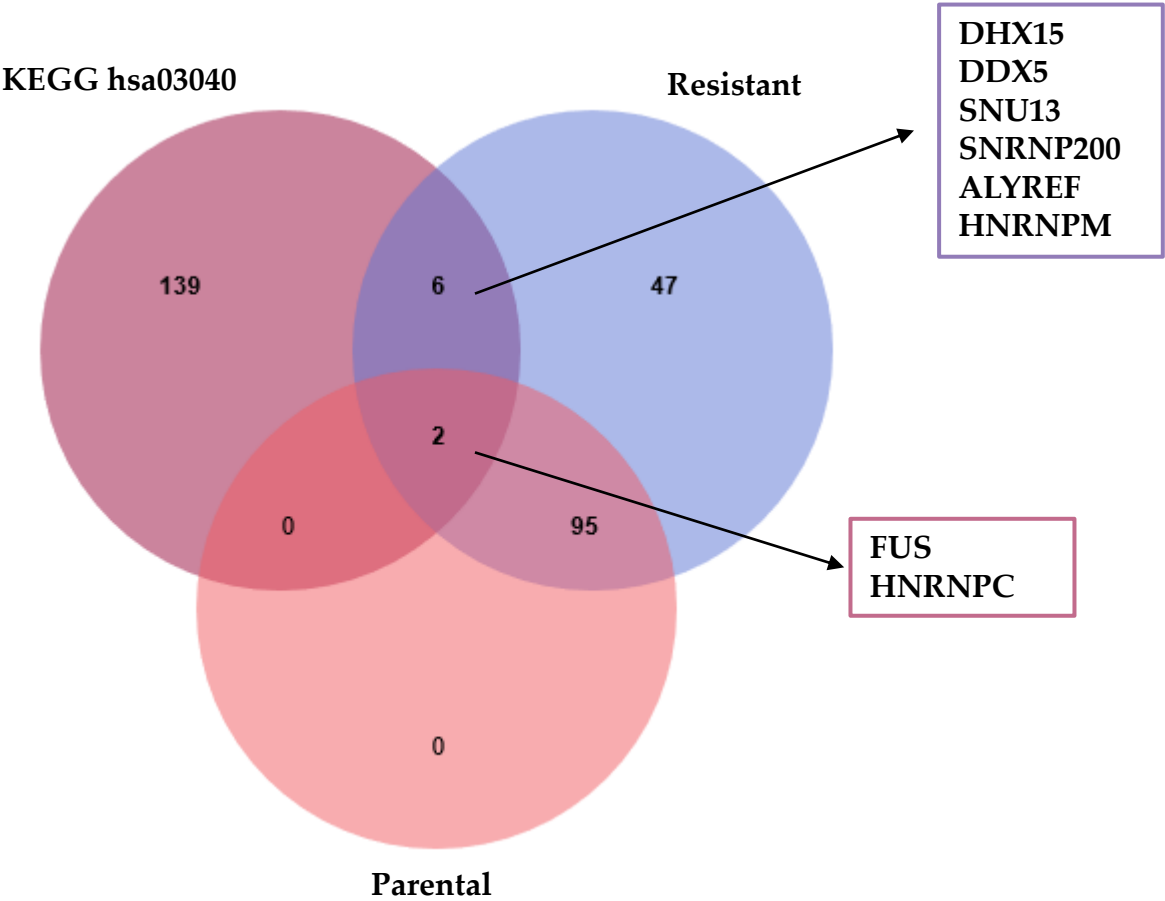

Figure S4

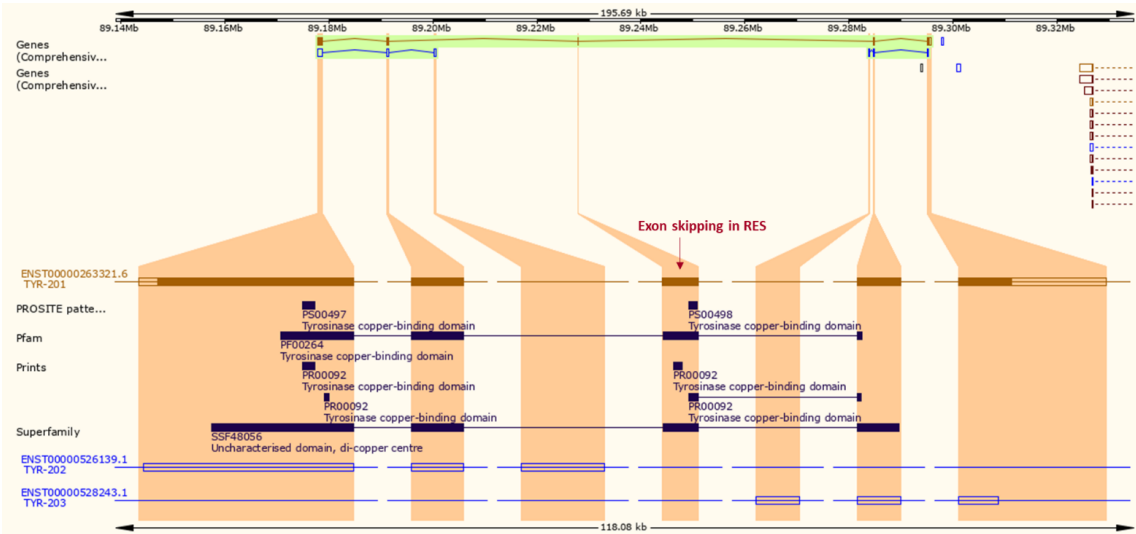

Figure S5

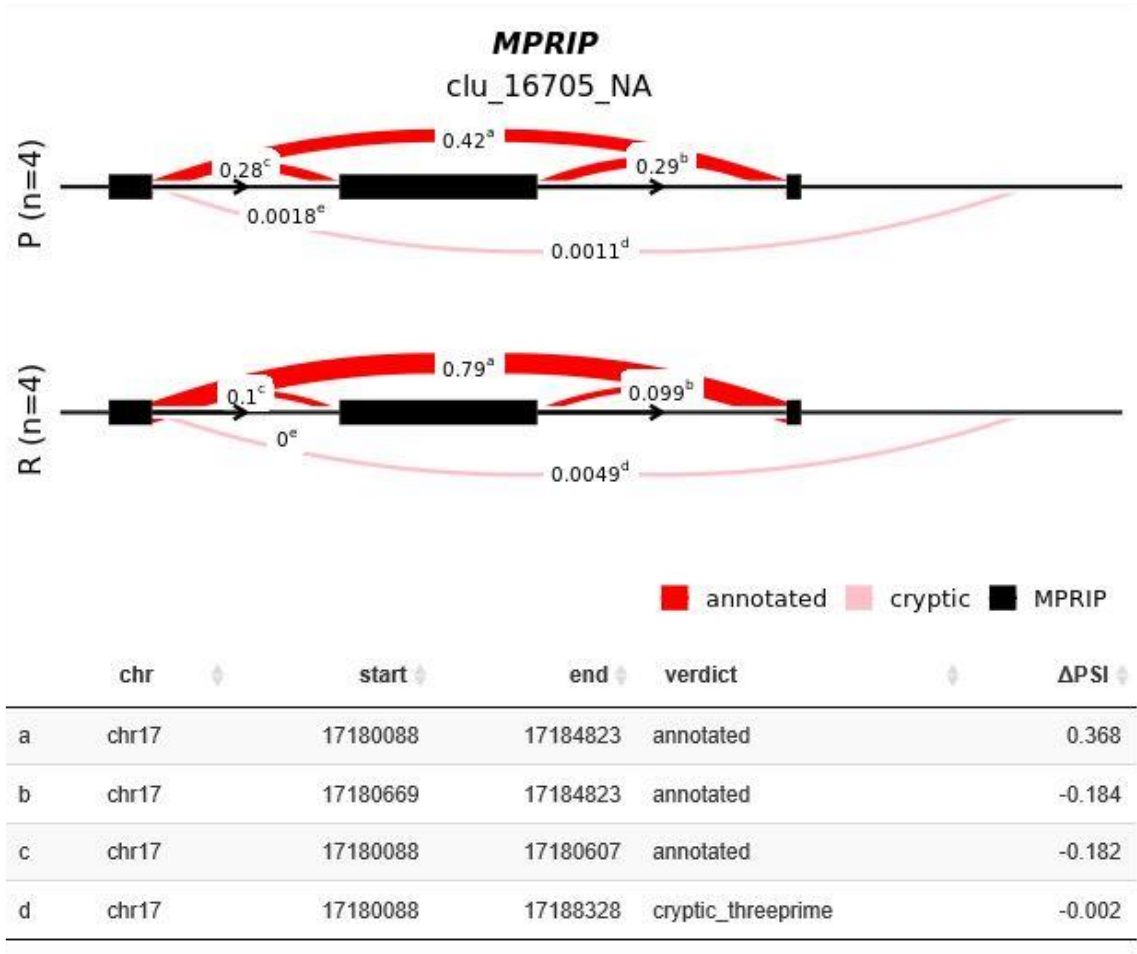

Supplement: Supplementary file 1 [file biomolecules-12-00993-s001.zip › Supplemental Figures.pdf]
